# Characterization of Non-coding DNA Satellites Associated with Sweepoviruses (Genus *Begomovirus*, *Geminiviridae*) – Definition of a Distinct Class of Begomovirus-Associated Satellites

**DOI:** 10.3389/fmicb.2016.00162

**Published:** 2016-02-17

**Authors:** Gloria Lozano, Helena P. Trenado, Elvira Fiallo-Olivé, Dorys Chirinos, Francis Geraud-Pouey, Rob W. Briddon, Jesús Navas-Castillo

**Affiliations:** ^1^Instituto de Hortofruticultura Subtropical y Mediterránea “La Mayora”, Universidad de Málaga – Consejo Superior de Investigaciones CientíficasAlgarrobo-Costa, Spain; ^2^Universidad del ZuliaMaracaibo, Venezuela; ^3^Agricultural Biotechnology Division, National Institute for Biotechnology and Genetic EngineeringFaisalabad, Pakistan

**Keywords:** *Begomovirus*, deltasatellites, DNA satellites, *Geminiviridae*, *Ipomoea*, *Merremia*, sweepoviruses, sweet potato

## Abstract

Begomoviruses (family *Geminiviridae*) are whitefly-transmitted, plant-infecting single-stranded DNA viruses that cause crop losses throughout the warmer parts of the World. Sweepoviruses are a phylogenetically distinct group of begomoviruses that infect plants of the family Convolvulaceae, including sweet potato (*Ipomoea batatas*). Two classes of subviral molecules are often associated with begomoviruses, particularly in the Old World; the betasatellites and the alphasatellites. An analysis of sweet potato and *Ipomoea indica* samples from Spain and *Merremia dissecta* samples from Venezuela identified small non-coding subviral molecules in association with several distinct sweepoviruses. The sequences of 18 clones were obtained and found to be structurally similar to tomato leaf curl virus-satellite (ToLCV-sat, the first DNA satellite identified in association with a begomovirus), with a region with significant sequence identity to the conserved region of betasatellites, an A-rich sequence, a predicted stem–loop structure containing the nonanucleotide TAATATTAC, and a second predicted stem–loop. These sweepovirus-associated satellites join an increasing number of ToLCV-sat-like non-coding satellites identified recently. Although sharing some features with betasatellites, evidence is provided to suggest that the ToLCV-sat-like satellites are distinct from betasatellites and should be considered a separate class of satellites, for which the collective name deltasatellites is proposed.

## Introduction

Conventional satellites are subviral agents which lack genes that encode functions needed for replication, depending instead for their multiplication on the co-infection of a host cell with a helper virus (Briddon et al., 2012). Two major classes of satellites may be distinguished, satellite viruses that encode a structural protein that encapsidates their genome, and satellite nucleic acids that encode either non-structural proteins, or are non-coding, and are encapsidated by the coat protein (CP) of helper viruses. The genetic material of satellites is distinct from that of the genome of their helper viruses. Replication of the satellites interferes with the replication of the helper virus and may affect disease symptoms, ranging from attenuation to exacerbation depending on the satellite, the helper virus and the host plant (Hull, 2002; Simon et al., 2004).

Viruses of the genus *Begomovirus* (family *Geminiviridae*) have circular single-stranded DNA genomes composed of one or two components each of ∼2.7 kb. They are transmitted exclusively by the whitefly *Bemisia tabaci* (Hemiptera: Aleyrodidae) and cause important diseases of dicotyledonous crops worldwide (Navas-Castillo et al., 2011). The sweepoviruses constitute a monophyletic group of begomoviruses which have been identified infecting sweet potato (*Ipomoea batatas*) and other species of the family Convolvulaceae. Typical of monopartite begomoviruses originating from the Old World (OW), the genomes of sweepoviruses encode two genes (the CP and V2) in the virion-sense and four [the replication associated protein (Rep), the transcriptional activator protein (TrAP), the replication-enhancer protein (REn) and the C4 protein] in the complementary-sense. Virion- and complementary-sense genes diverge from an intergenic region (IR) that contains a predicted stem–loop structure with the conserved (between most geminiviruses) nonanucleotide sequence TAATATTAC forming part of the loop. The IR also contains repeated short sequence motifs (known as iterons) surrounding the TATA box of the Rep promoter that are Rep binding sites and, together with the stem–loop structure, form the origin of virion-sense DNA replication (*ori*). The sweepoviruses cluster as a branch in the begomovirus phylogenetic tree below the divergence between the OW and New World (NW) branches, corresponding to the major clades in the genus (Lozano et al., 2009).

Two classes of circular single-stranded DNA molecules have been described in association with geminiviruses, the alphasatellites and the betasatellites (Briddon and Stanley, 2006; Zhou, 2013). The alphasatellites (previously known as DNA-1; Briddon et al., 2004) are not strict satellites, since they are capable of autonomous-replication in plant cells, but are dependent on their helper begomoviruses for movement within plants and insect transmission between plants (Mansoor et al., 1999; Saunders and Stanley, 1999). Alphasatellites are widespread in the OW and usually occur in association with monopartite begomoviruses and betasatellites. Alphasatellites have also been identified in the NW in association with bipartite begomoviruses and in the absence of betasatellites (Paprotka et al., 2010b; Romay et al., 2010). Recently an alphasatellite and a betasatellite were shown to be associated with a mastrevirus (genus *Mastrevirus*, family *Geminiviridae*) in wheat, the first time either of these components has been identified in a monocot (Kumar et al., 2014).

Betasatellites (previously known as DNA-β) have so far only been identified in the OW and are usually, but not exclusively, associated with monopartite begomoviruses (Saunders et al., 2000; Briddon et al., 2001). They are also half the size of begomovirus components (∼1360 nt) and contain three conserved regions: an A-rich sequence, a sequence conserved between all betasatellites known as the satellite conserved region (SCR) and a single gene in the complementary-sense that encodes a small protein (∼118 amino acids) known as βC1. Betasatellites increase the accumulation of their helper begomoviruses and enhance the symptoms induced in some host plants (Briddon et al., 2003; Zhou et al., 2003), likely due to the suppressor of RNA interference activity of the βC1 protein (Cui et al., 2005; Yang et al., 2011). The only sequence similarity between betasatellites and their helper begomoviruses is the nonanucleotide sequence TAATATTAC within the stem–loop structure forming part of the SCR. Betasatellites and alphasatellites are promiscuous with respect to helper virus in that they may be maintained in plants by several, but possibly not all, geminiviruses (Saunders et al., 2002a,b, 2008; Briddon et al., 2003).

The first DNA satellite was identified in association with a plant virus in tomato plants infected with the OW monopartite begomovirus tomato leaf curl virus (ToLCV) originating from Australia (Dry et al., 1997). ToLCV-sat is 682 nt, about one quarter the size of a begomovirus genome/genomic component, is non-coding and has no significance sequence similarity with the helper virus. The satellite contains a predicted stem–loop structure containing the geminivirus-like nonanucleotide sequence TAATATTAC, a sequence with similarity to the SCR of betasatellites, an A-rich sequence and a second predicted stem–loop structure that contains a sequence identical to the predicted iteron sequence of ToLCV within the loop. In common with betasatellites, ToLCV-sat depends upon a helper virus for replication and, based upon sequence and structural similarities with betasatellites, it is believed that ToLCV-sat originated as a defective betasatellite (Saunders et al., 2000).

A novel class of DNA satellites has recently been identified in association with begomoviruses infecting malvaceous plants in Cuba (Fiallo-Olivé et al., 2012). These satellites are approximately one quarter the size of a begomovirus genome/genomic component and have all the features of ToLCV-sat; containing a stem–loop structure with the nonanucleotide TAATATTAC forming part of the loop, an A-rich region and are non-coding. They also contain a putative second predicted stem–loop structure situated close to begomovirus iteron-like sequences and a TATA motif, and a short region with sequence identity to the SCR of betasatellites. Similar satellites have also been identified using vector-enabled metagenomics (VEM) in *B. tabaci* adults collected in Florida (Ng et al., 2011).

Although an increasing number of sweepovirus species have been described in the last few years infecting sweet potato crops worldwide (Luan et al., 2007; Lozano et al., 2009; Albuquerque et al., 2011, 2012; Wasswa et al., 2011), only recently have satellites been shown in association with a sweepovirus. Geetanjali et al. (2013) showed the association of croton yellow vein mosaic betasatellite and papaya leaf curl betasatellite with sweet potato leaf curl virus (SPLCV) infecting *Ipomoea purpurea* in India. The study presented here has identified ToLCV-sat-like DNA satellites in association with sweepoviruses originating from Spain and Venezuela. On the basis of these findings it is proposed that the small non-coding DNA satellites associated with begomoviruses be considered a class of satellites distinct from betasatellites.

## Materials and Methods

### Plant Material and DNA Extraction

Leaf samples from 25 sweet potato (*I. batatas*) and three *Ipomoea indica* plants collected in Spain, previously shown to be infected with sweepoviruses, were analyzed (**Table 1**). Total nucleic acids were extracted from 0.1 g leaf tissue following the protocol of Crespi et al. (1991). Also, one sample of *Merremia dissecta* collected in Sucre, Venezuela, showing typical begomovirus symptoms that included leaf mosaic was analyzed after DNA extraction from dried leaf material using a CTAB-based purification method (Haible et al., 2006).

**Table 1:** Sweet potato (*Ipomoea batatas*), *I. indica* and *Merremia dissecta* samples analyzed in this work.

Host	Origin	Year	Sample	Presence of DNA satellites	Presence of begomoviruses
*I. batatas*	Málaga (ES^a^)	2002	B0	-	SPLCV^d,f^
	Málaga (ES)	2002	B2	-	SPLCV^f^
	Málaga (ES)	2002	B3	+	SPLCV^f^
	Málaga (ES)	2002	B5	-	SPLCV^f^
	Málaga (ES)	2002	B6	-	SPLCV^f^
	Málaga (ES)	2002	B9	-	SPLCV^f^
	Málaga (ES)	2002	B10	-	SPLCV^f^
	Málaga (ES)	2002	B11	-	SPLCV^f^
	Málaga (ES)	2002	B12	+	SPLCV^f^
	Málaga (ES)	2002	B13	-	SPLCV^f^
	Málaga (ES)	2002	B16	+	SPLCV^f^
	Málaga (ES)	2002	B17	-	SPLCV^f^
	Málaga (ES)	2002	B18	-	SPLCV^f^
	Málaga (ES)	2002	B19	-	SPLCV^f^
	Málaga (ES)	2006	270906/1d	-	SPLCV^g^
	Málaga (ES)	2006	270906/4b	-	SPLCV^g^
	Málaga (ES)	2006	270906/5b	-	SPLCV^g^
	Málaga (ES)	2006	270906/5c	-	SPLCV^g^
	Málaga (ES)	2006	270906/6b	-	SPLCV^g^
	Málaga (ES)	2006	270906/6d	-	SPLCV^g^
	Tenerife (CI^b^, ES)	2002	B25	-	SPLCV^f^
	Tenerife (CI, ES)	2002	B29	-	SPLCV^f^
	Lanzarote (CI, ES)	2002	B32	+	SPLCV, SPLCCaV^e,h^
	Lanzarote (CI, ES)	2002	B33	-	SPLCV^f^
	Lanzarote (CI, ES)	2002	B34	+	SPLCV^f^
*I. indica*	Málaga (ES)	2006	270906/3c	+	SPLCV^g^
	Málaga (ES)	2006	270906/3d	+	SPLCV^g^
	Málaga (ES)	2006	270906/3f	-	SPLCV^g^
*M. dissecta*	Sucre (VE^c^)	2009	1764	+	SPLCV^i^


*^a^ES, Spain; ^b^CI, Canary Islands; ^c^VE, Venezuela; ^d^SPLCV, sweet potato leaf curl virus; ^e^SPLCCaV, sweet potato leaf curl Canary virus; ^f^Lozano, 2007; ^g^Trenado, 2009; ^h^Lozano et al., 2009; ^i^This work.*

### Amplification and Cloning of Subviral DNA Molecules

A pair of primers previously designed to amplify the full-length genomic components of all sweepoviruses identified in Spain (MA369 5′-GGGAATGCTGTCCCAATTGCTGC-3′, MA370 5′-AGTGTAAGGCACGATAGCTGTCTC-3′; Lozano et al., 2009) was used to amplify and clone a chimeric molecule (pBG9) from sample B32. A pair of abutting primers was designed to the non-viral sequence of pBG9 to PCR amplify putative circular subviral DNA molecules associated to the sweepoviruses present in the 28 sweet potato and *I. indica* samples: MA465 (5′-CCTTAGCTTCGCACGTAGCTA-3′) and MA466 (5′-CTGCTTAGCGTAGCGGTTTGG-3′). PCR was carried out with Expand High Fidelity DNA polymerase (Roche Diagnostics, Mannheim, Germany) using the program: denaturation for 2 min at 94°C; 10 cycles of denaturation for 15 s at 94°C, hybridization for 30 s at 50°C and extension for 90 s at 72°C; 20 cycles of denaturation for 15 s at 94°C, hybridization for 30 s at 50°C and extension for 90 s at 72°C, adding 5 s of extension per cycle; followed by a final extension step of 7 min at 72°C. The DNA fragments amplified by PCR were purified using the High Pure PCR Product Purification Kit (Roche Diagnostics) and cloned in pGEM-T Easy Vector (Promega, Madison, WI, USA).

DNA extracted from the *M. dissecta* sample was amplified by rolling-circle amplification (RCA) with φ29 DNA polymerase using the TempliPhi DNA Amplification Kit (GE Healthcare, Little Chalfont, UK). The RCA product was digested with *Eco*RI and cloned in an *Eco*RI digested, covalently closed pGEM-T-Easy Vector (Promega). Also, a *Bam*HI fragment of 586 bp corresponding to a partial sequence of a sweepovirus genome was cloned in pBSK+ after RCA from the same sample. A pair of abutting primers (MA1402 5′-catgGAGCTCTGTACGGAGTTAATCCGTATATTC-3′, MA1403 5′-catgGAGCTCGAACCCTAGGGTTCCTGGC-3′; *Sac*I restriction site present in the sweepovirus genome used for subsequent cloning is underlined and nucleotides added to facilitate *Sac*I digestion are in lower case letters) was designed based on this sequence to amplify by PCR the complete sweepovirus genome present in this sample. PCR was carried out with BioTaq DNA polymerase (Bioline, London, UK) using the program: denaturation for 2 min at 94°C; 30 cycles of denaturation for 1 min at 94°C, hybridization for 1 min at 55°C, and extension for 3 min at 72°C; followed by a final extension step of 5 min at 72°C. The ∼2.8 kbp DNA fragment amplified from the *M. dissecta* sample using the RCA product as the template, was digested with *Sac*I and cloned in a covalently closed pGEM-T-Easy Vector (Promega).

The possible presence of alphasatellites and betasatellites in samples was examined by PCR using primers DNA101/DNA102, UN101/UN102, and beta01/beta02 as previously reported (Briddon et al., 2002; Bull et al., 2003).

### Southern Blot Analysis

The presence of DNA satellites in one of the sweet potato samples (B3) was assessed by Southern blot hybridization. Total DNA (∼1 μg) was separated on 0.8% agarose gel electrophoresis in Tris-acetic acid-EDTA buffer (TAE), transferred to a positively charged nylon membrane (Roche Diagnostics) using vacuum (GE Healthcare) and fixed with ultraviolet light (Crosslinker RPN 2500, Amersham Life Science, Friburg, Germany). DNA was then hybridized [65°C in standard buffer (Roche Diagnostics) with 50% formamide] with a digoxigenin-labeled probe synthesized by PCR from clone pSBG53 (**Table 2**). For this, the insert of pSBG53 was released by digestion with *Eco*RI and amplified with primers MA465 and MA466. DIG-dUTP was incorporated by using the PCR DIG Probe Synthesis System Kit (Roche Diagnostics) according to the manufacturer’s instructions. As a size control, Southern blot analysis was also carried out with the same sample using a probe corresponding to the coat protein gene of SPLCV, isolate BG30 (Trenado et al., 2011). For this, DNA was analyzed both undigested and digested with a restriction enzyme which recognizes a unique site in the satellite (*Pst*I) and SPLCV (*Bam*HI).

**Table 2:** Sweepovirus-associated DNA satellites cloned in this work.

Clone	Sample	Size (nt)	GenBank acc. no.
SBG32	B32	662	FJ914391
SBG51	B32	662	FJ914390
SBG52	B3	664	FJ914392
SBG53	B3	707	FJ914393
SBG54	B12	708	FJ914394
SBG55	B12	707	FJ914395
SBG56	B16	694	FJ914396
SBG57	B34	750	FJ914397
SBG58	B34	662	FJ914398
SBG59	B32	738	FJ914403
SBGB3-5	B3	633	FJ914404
SBGB3-6	B3	707	FJ914405
SI3C-3	270906/3c	694	FJ914399
SI3C-5	270906/3c	704	FJ914400
SI3D-11	270906/3d	705	FJ914401
SI3D-12	270906/3d	705	FJ914402
1764E13	1764	733	KF716173
1764E34	1764	733	KF716174


### Sequencing and Bioinformatic Analysis

The sequences of the cloned inserts were obtained with an automatic sequencer (Secugen, Madrid, Spain or Macrogen, Inc., Seoul, South Korea). SeqMan, part of the Lasergene sequence analysis package (DNAStar, Inc., Madison, WI, USA), was used to assemble the sequences. BLAST^1^ was used for sequence similarity searches of the GenBank database. Sweepovirus and satellite sequences were aligned with MUSCLE (Edgar, 2004) and pairwise identity scores were calculated with the Species Demarcation Tool (SDT) 5 (Muhire et al., 2014). MEGA 6 (Tamura et al., 2013) was used for phylogenetic inference by the Neighbor–Joining method. The search for A-rich regions in the satellite molecules was carried out using the online DNA base composition analysis tool^2^. The sequences used in phylogenetic analyses and their accession numbers in GenBank are shown in **Supplementary Table 1**. The ToLCV-sat nucleotide sequence was randomly shuffled and used as an outgroup^3^ (Stothard, 2000).

## Results

### Detection of a Virus-Satellite Chimera in a Sweet Potato Sample

**FIGURE 1:**
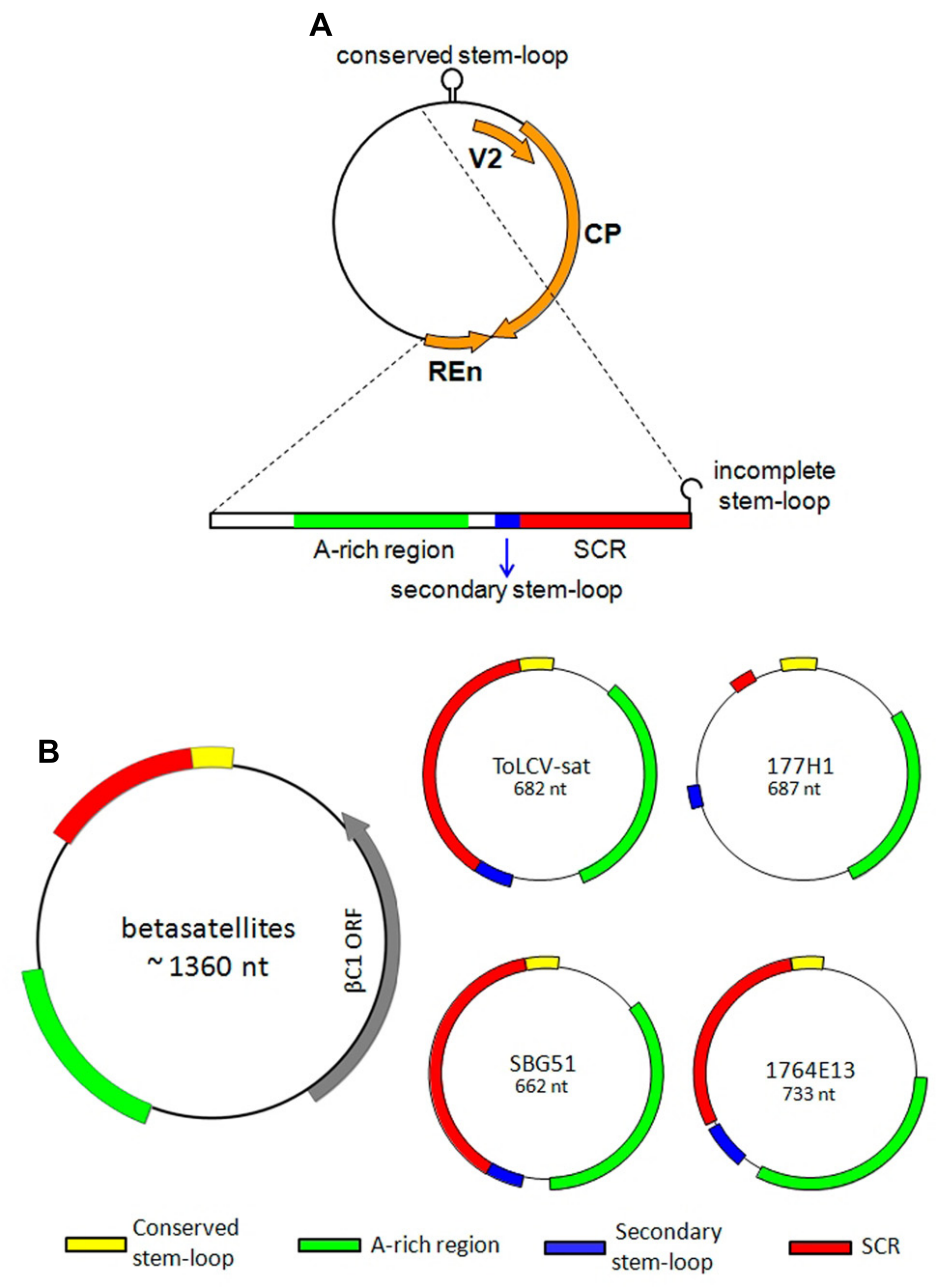
**Schematic representation of the chimeric sweepovirus-DNA satellite molecule identified in sweet potato sample B32 **(A)** and representative deltasatellites **(B)**.** The virus genes/gene fragments of the chimeric molecule are the V2 gene, coat protein (CP) gene and replication-enhancer (REn) gene. Clones SBG51 and 1764E13 represent DNA satellites associated with *Ipomoea*- and *Merremia*-infecting sweepoviruses, respectively. ToLCV-sat and 177H1 are small DNA satellites that have been described previously (Dry et al., 1997; Fiallo-Olivé et al., 2012). The main features of these molecules include the conserved stem–loop, a secondary stem–loop, the satellite conserved region (SCR) and an A-rich region. A schematic representation of a betasatellite is included for comparison.

During a survey for the presence of sweepoviruses in sweet potato samples from Spain, using the abutting primers MA369 and MA370, a clone with an unexpected sequence was obtained from sample B32 (collected in Lanzarote, Canary Islands, in 2002; Lozano et al., 2009). The insert of this clone (pBG9) was determined to be 2598 bp in length (acc. no. EF591125) and a BLAST search showed significant identity with available sequences of sweepoviruses for only about 70% of the length (coordinates 2240-1451). This region contains the V2 and CP genes, a truncated REn gene lacking the first 71 nt, and the complete IR including the virus iterons, thus allowing transreplication (**Figure 1A**). This fragment showed 99.8% identity to the corresponding region of the genome of the sweepovirus sweet potato leaf curl Canary virus (SPLCCaV) that was isolated from the same sample [isolates BG21 (EU856365) and BG25 (FJ529203); Lozano et al., 2009]. BLAST analysis of the remaining 788 bp (coordinates 1452-2239) showed significant levels of identity with ToLCV-sat (U74627). Alignment of the non-sweepovirus region of pBG9 and ToLCV-sat showed a nucleotide identity of 74.0%. This DNA fragment contains several features in common with ToLCV-sat, consisting of an A-rich region (154 nt, containing 61.0% A), a region with significant identity with the SCR of betasatellites, a stem–loop structure containing an incomplete nonanucleotide, and a second predicted stem–loop structure (**Figure 1A**).

### Small Circular DNA Satellites are Frequently Associated with Sweepoviruses Infecting Sweet Potato and *I. indica* in Spain

A pair of abutting primers (MA465, MA466) was designed to the satellite-like sequence contained in the chimeric clone pBG9. PCR amplification using primers MA465/MA466 yielded a DNA fragment of ∼700 bp for 7 out of 28 samples from Spain; five sweet potato plants from Málaga and Lanzarote (Canary Islands) and two *I. indica* plants from Málaga (**Table 1**). These samples have been previously shown to be infected by the sweepovirus SPLCV and sample B32 was infected in addition with SPLCCaV (**Table 1**). The sequences of 16 clones were obtained and these ranged in size from 633 to 750 nt (**Table 2**). The overall nucleotide identity between the 16 clones ranged from 94.6 to 99.9%, and with the satellite-derived sequence of pBG9 ranged from 93.2 to 98.9%.

**FIGURE 2:**
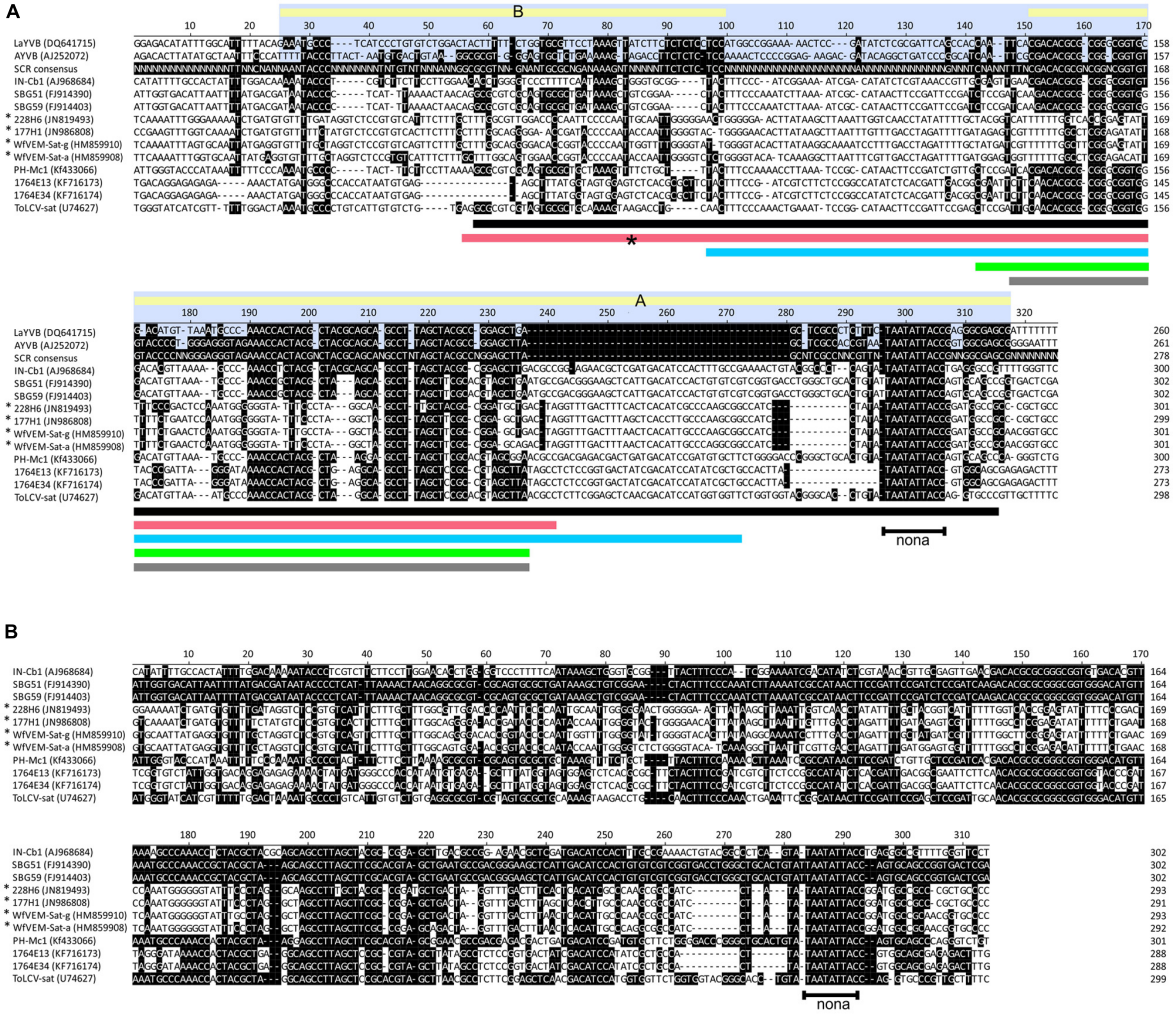
**Sequences of the small satellites homologous to the satellite conserved region of betasatellites.** **(A)** Alignment of the sequences immediately upstream of the nonanucleotide (nona)-containing hairpin (at coordinate 300 of the alignment) of two sweepovirus-associated satellites from Spain (SBG59 and SBG51), two sweepovirus-associated satellites from Venezuela (1764E13 and 1764E34), two satellites from Cuba (177H1 and 228H6; Fiallo-Olivé et al., 2012), two satellites isolated from whiteflies from Florida (WfVEM-Sat-a and WfVEM-Sat-g; Ng et al., 2011), the tomato leaf curl virus-satellite (ToLCV-sat; Dry et al., 1997), a small satellite isolated from *C. bonplandianus* originating from India (IN-Cb-1), a small satellite isolated from *Malvastrum coromandelianum* originating from the Philippines (PH-Mc-1) and two betasatellites [*Ageratum* yellow vein betasatellite (AYVB); Saunders et al., 2000] and *Lindernia anagallis* yellow vein betasatellite (LaYVB; Ha et al., 2008). For each the database accession number is given. NW satellites from Cuba and Florida are highlighted with asterisks. The approximate extent of the SCR of betasatellites is delimited by the blue highlighting. This contains two regions of conserved sequence (indicated by the yellow bars labeled A and B) separated by a region of sequence that is not conserved. The betasatellite SCR consensus sequence was produced from an alignment of 158 betasatellites [all full-length betasatellites available in the nucleotide sequence databases (sampled June 2014), but excluding those produced using primers beta01/beta02] using the program “cons,” part of the EMBOSS suite of programs (Rice et al., 2000). Shown are nucleotide sequence positions with greater than 50% conservation. The colored bars below each block of the alignment are discussed in the text. **(B)** Alignment of the sequences immediately upstream of the nonanucleotide (nona) containing hairpin (at coordinate 285 of the alignment) of small satellites. Nucleotide sequences with identity to the sequence of SBG51 are highlighted by white text on a black background.

A BLAST analysis of the sequences against the GenBank nucleotide sequence database identified sequence relatedness with a small molecule (673 nt in length) isolated from a begomovirus infected *Malvastrum coromandelianum* plant originating from the Philippines, which will henceforth be referred to as PH-Mc1 (KC577540; 86% identity across ∼227 nt; black bar in **Figure 2A**) and an ∼198 nt fragment of ToLCV-sat (U74627, 84% identity, red bar in **Figure 2A**). Also, BLAST analysis identified additional sequence relatedness with an ∼162 nt fragment of a small molecule (739 nt in length) isolated from a begomovirus infected *Croton bonplandianus* plant originating from India which will henceforth be referred to as IN-Cb1 (AJ968684, 76% identity, blue bar in **Figure 2A**) and an approximately 80 nt fragment of a number of betasatellites, with the highest levels identity (89%) to *Lindernia anagallis* yellow vein betasatellite (LaYVB) originating from Vietnam (DQ641715; Ha et al., 2008; gray bar in **Figure 2A**).

**FIGURE 3:**
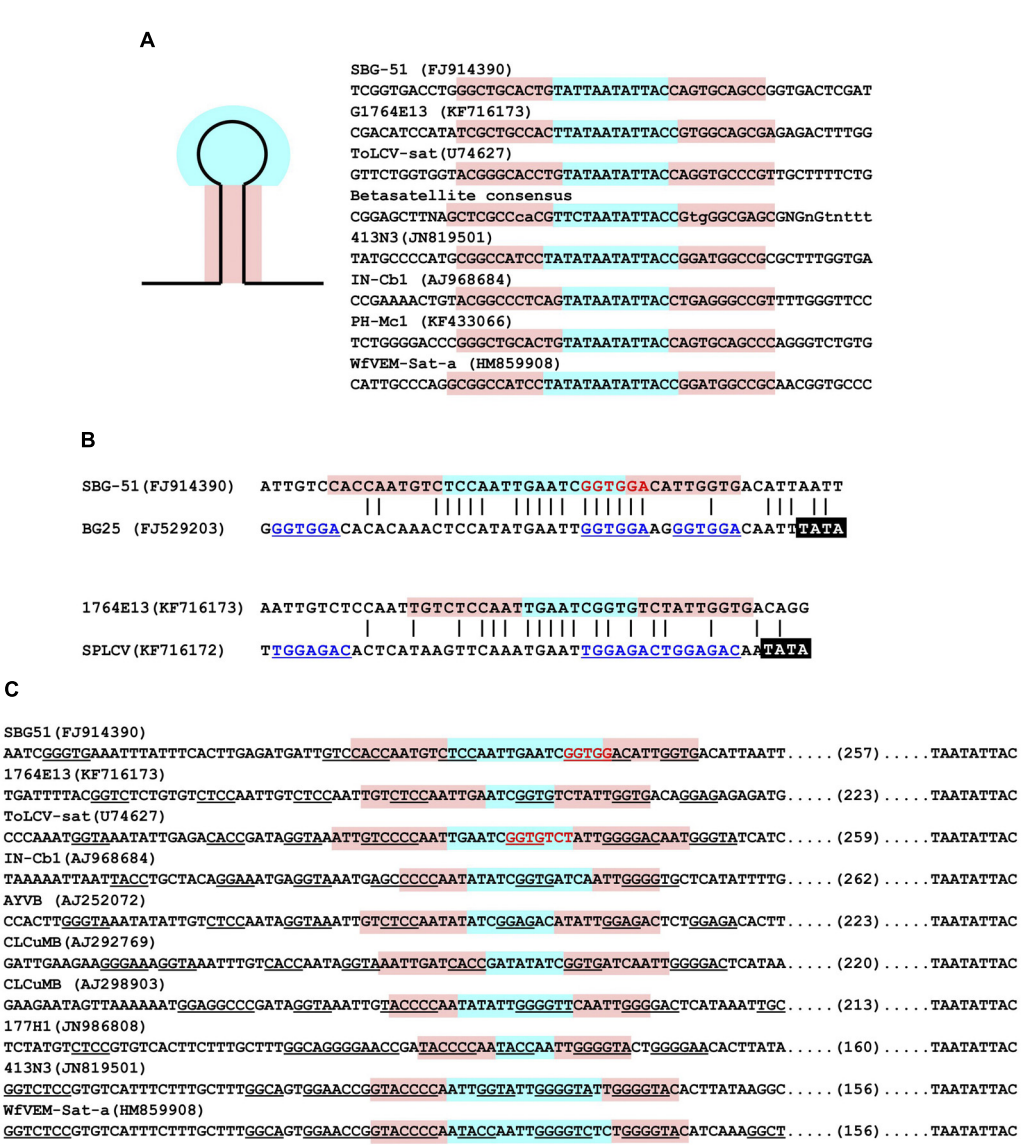
**Hairpin structures encoded by small begomovirus-associated satellites.** **(A)** Comparisons of the sequences encompassing the nonanucleotide (TAATATTAC) containing hairpin structures of selected small satellites and the consensus sequence (produced as described for **Figure 2**) of betasatellites. **(B)** Alignment of the secondary hairpin structures of the satellites SBG51 and 1764E13 with the iteron containing sequences of their helper begomoviruses (sweet potato leaf curl virus). The TATA box of the Rep promoter is highlighted in each case by white text on a black background. The predicted iteron sequences of the virus are underlined in each case. The sequence of satellite SBG-51 with identity to the iteron sequence of the helper virus is highlighted in red text. **(C)** Comparisons of the sequences encompassing the secondary hairpin structures of selected satellites, including three betasatellite sequences [cotton leaf curl Multan betasatellite (CLCuMB) and *Ageratum* yellow vein betasatellite (AYVB)]. The sequence of satellite SBG-51 and ToLCV-sat with identity to the predicted iteron sequences of the respective helper virus is highlighted in red text. Iteron-like sequences, in both the forward and reverse orientations, are underlined. Iteron-like sequences are here defined as any four nucleotides beginning GG, since this dinucleotide sequence frequently forms part of iterons (Argüello-Astorga and Ruiz-Medrano, 2001). For each panel the loop of the predicted hairpin is highlighted in blue and the two legs of the stem in pink.

The sequences of the 16 clones were found to be structurally similar to ToLCV-sat, with a region with significant sequence identity to the SCR of betasatellites, an A-rich sequence, a predicted stem–loop structure containing the nonanucleotide TAATATTAC (**Figures 1B** and **3A**), and a second predicted stem–loop (**Figures 1B** and **3C**). With the exception of the nonanucleotide, the sequences of these molecules are unrelated to the sequences of begomoviruses. The differences in sizes of the molecules is due mainly to the presence/absence of a 70–90 nt duplicated region located immediately downstream of the nonanucleotide-containing hairpin structure, duplicating the 3′ leg of the hairpin, and an insertion/deletion of 29–31 nt in the A-rich sequences [coordinates 339–368 for pSBG57 (FJ914397)].

An analysis for potential coding regions indicated the presence of a number of small ORFs, all with a predicted coding capacity of less than 50 amino acids, but with no obvious RNA polymerase II promoter elements required to initiate transcription. This suggests that the molecules are non-coding. The A-rich regions are 190–234 nt in length with 51.5–53.9% adenine content. The sequences with similarity to the SCR of betasatellites, determined following the limits defined by Briddon et al. (2003), are ∼254 nt in length (with the exception of that of SBG52 that is composed of ∼233 nt) and the identity between them ranged from 95.7 to 100%.

A second predicted stem–loop structure is located between the A-rich region and the SCR-like sequences, as described for ToLCV-sat and satellites associated with bipartite NW begomoviruses infecting malvaceous hosts in the Caribbean (**Figure 3C**). The loop contains a sequence identical to the predicted iterons (Rep-binding sequences) of sweepoviruses and some sequence similarity to the Rep binding domain that lies just upstream of the TATA box of the Rep promoter (**Figure 3B**).

**FIGURE 4:**
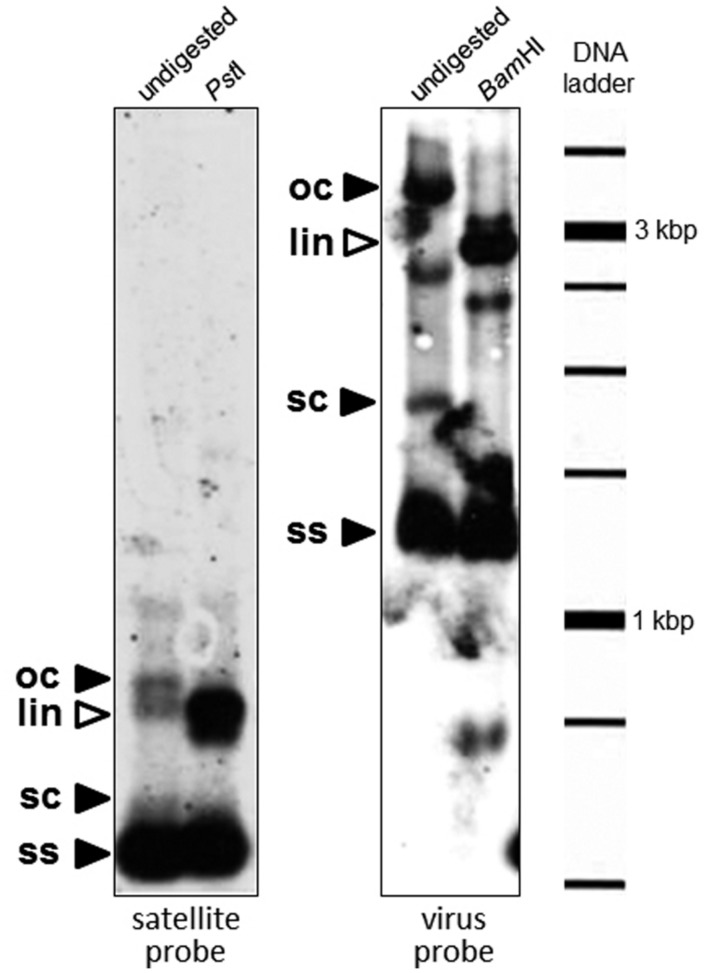
**Southern blot analysis to detect the presence of DNA satellites in a sweepovirus-infected sweet potato sample (B3).** Total DNA (∼1 μg) was separated on 0.8% agarose gel electrophoresis in TAE, transferred to a positively charged nylon membrane and hybridized with digoxigenin-labeled probes synthesized by PCR from clone SBG53 (satellite, left panel) and the sweet potato leaf curl virus (SPLCV) isolate present in that sample (virus, right panel). Total DNA was digested (right part of each panel) with a restriction enzyme which recognizes a unique site in the satellite (*Pst*I) and SPLCV (*Bam*HI). The positions of open circular (oc), supercoiled (sc), and single-stranded (ss) DNA forms are indicated with black arrowheads. The positions of linear (lin) DNA forms are indicated with white arrowheads. Mobility of the size marker (1 kb DNA ladder) is given in the right margin.

A Southern blot analysis of DNA extracted from sweet potato plant B3, probed with SBG53, detected the DNA forms typical of rolling-circle replication (**Figure 4**). There was no hybridization of this probe with total nucleic acid extracted from apparently healthy sweet potato plants in which no sweepoviruses or satellites were detected by PCR. Additionally, for none of the *Ipomoea* samples analyzed was amplification evident in PCR with primers DNA101/DNA102, UN101/UN102 or beta01/beta02, indicating the absence of alphasatellites and betasatellites.

### Distinct DNA Satellites are also Associated with a Sweepovirus Infecting *Merremia dissecta* in Venezuela

Digestion of the RCA product, obtained from the *M. dissecta* sample, with *Eco*RI resulted in a DNA product of about 750 bp. Cloning and sequencing of the inserts from four *Eco*RI clones showed all to be 733 bp in length with three having identical nucleotide sequences, represented by clone pG1764E13 (acc. no. KF716173). The fourth clone, pG1764E34 (KF716174), differed by only one nucleotide from pG1764E13.

BLAST analysis of the full-length satellite sequences from *Merremia* showed the highest percentage identity (65%) across only 35% of the sequence with ToLCV-sat. This sequence spans the SCR-like sequences of ToLCV-sat but extends further upstream than the SCR sequences of betasatellites. A BLAST analysis additionally identified sequence identity (79%) with a large number of betasatellites but spanning only 10% or less of the sequence within the SCR of betasatellites (gray bar in **Figure 2A**).

A more detailed sequence analysis showed a structure for the clones obtained from *Merremia* to be identical to that of ToLCV-sat and the satellites associated with the sweepoviruses infecting *Ipomoea* sp. in Spain; consisting of an A-rich region, a region with significant levels of identity with the SCR of betasatellites and, in addition to the conserved nonanucleotide-containing stem–loop structure, a second predicted stem–loop (**Figure 1B**). Although, the sequence of the second stem–loop structure contains some levels of sequence identity to the iteron-containing Rep-binding domain of the sweepovirus present in the same sample [this sweepovirus was cloned using a pair of abutting primers designed on a 806 bp clone obtained after RCA and digestion with *Bam*HI, and was shown to be an isolate of SPLCV (KF716172)], unlike ToLCV-sat and the satellites in sweet potato and *I. indica*, this lacks the predicted iterons sequences of the virus (**Figure 3B**).

Analysis of the *Merremia* satellites for potential coding sequences indicated the presence of small ORFs, the longest with a predicted coding capacity of 53 aa, with no obvious RNA polymerase II promoter elements required to initiate transcription. As for the *Ipomoea* samples, PCR amplification with primers DNA101/DNA102, UN101/UN102, or beta01/beta02 did not result in products, indicating the absence of alphasatellites and betasatellites.

### Relationships of the Small DNA Satellites Associated with Begomoviruses

**FIGURE 5:**
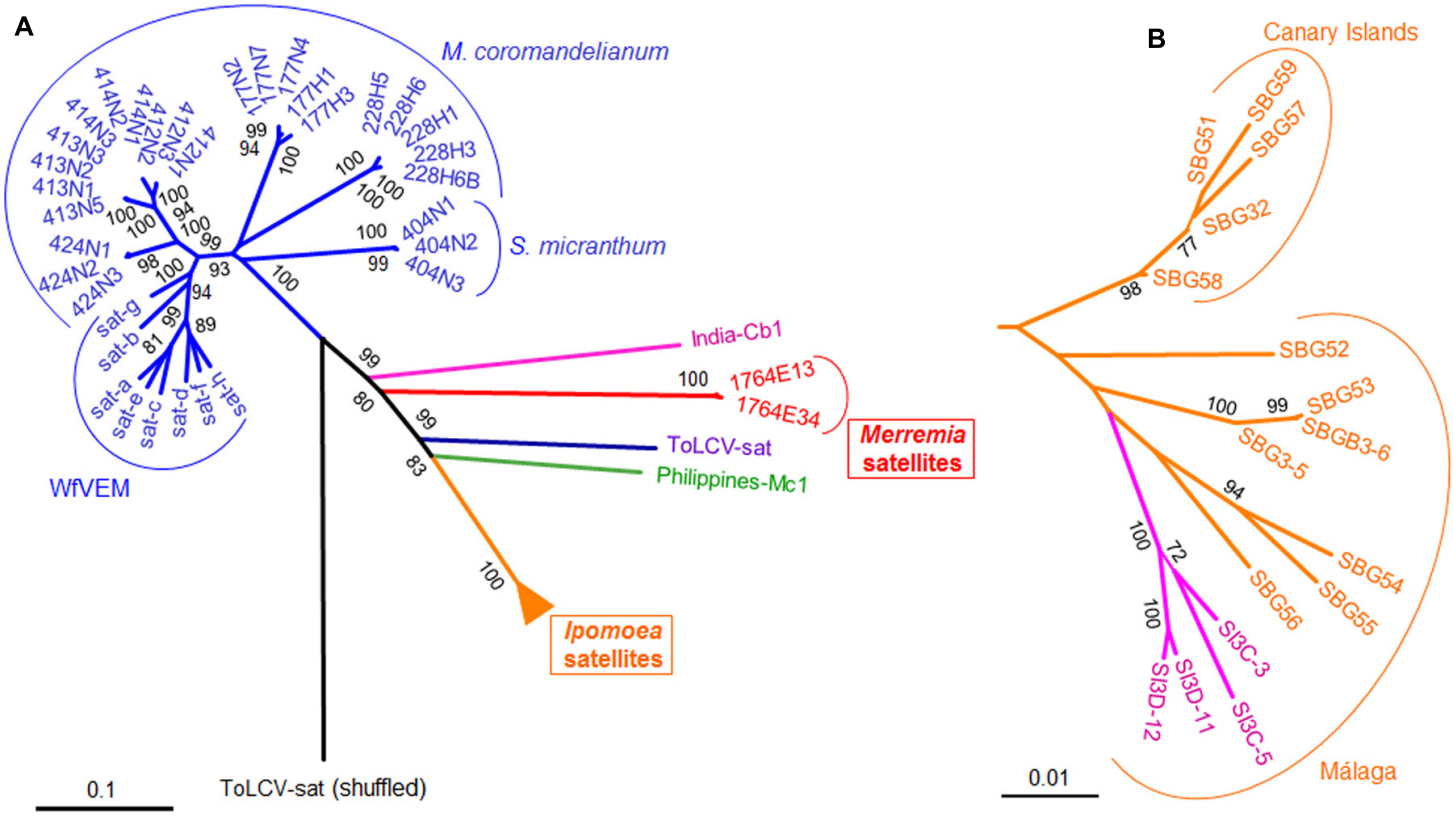
**Phylogenetic tree showing the relationships among the novel satellite molecules described in this work (*Ipomoea* and *Merremia* satellites) and all other deltasatellites.** Sequences retrieved from GenBank include small satellites associated with New World begomoviruses infecting Malvaceae, ToLCV-sat, a satellite isolated from *Croton bonplandianus* in India (India-Cb1), and a satellite isolated from *Malvastrum coromandelianum* in Philippines (Philippines-Mc1). The tree was constructed using the Neighbor-Joining method with MEGA 6. Only bootstrap values higher than 70% are shown. The *Ipomoea* satellite sub-tree, compressed in **(A)**, is expanded in **(B)**. In **(B)**, SB stands for *I. batatas* satellites (in orange) and SI stands for *I. indica* satellites (in purple). The ToLCV-sat nucleotide sequence was randomly shuffled and used as an outgroup. See **Table 2** and **Supplementary Table 1** for details on the analyzed sequences.

The sequences of the DNA satellites associated with sweepoviruses were aligned with other small DNA satellites and a phylogenetic tree was produced (**Figure 5**). The tree distinguishes three groups of sequences; (I) the sweepovirus-associated satellites from Spain, (II) the NW satellites (excluding the satellites from *Merremia*), and (III) all the other small satellites. The relationship between the groups, and the relationship within the third groups of satellites of diverse origins, is not well-defined, likely due to the low levels of sequence similarity. Within the *Ipomoea* sweepovirus-associated satellite cluster, the sequences group according to geographic origin (either mainland Spain or the Canary Islands), suggesting that the two groups of satellites have been isolated for some time and are evolving independently (**Figure 5B**). Similarly, within the satellites from mainland Spain, the isolates from *I. indica* form a single cluster but are more closely related to satellites from the mainland occurring in *I. batatas* than satellites occurring in *I. batatas* on the Canary Islands. This suggests that there is exchange, likely by whitefly transmission, between these two hosts.

**FIGURE 6:**
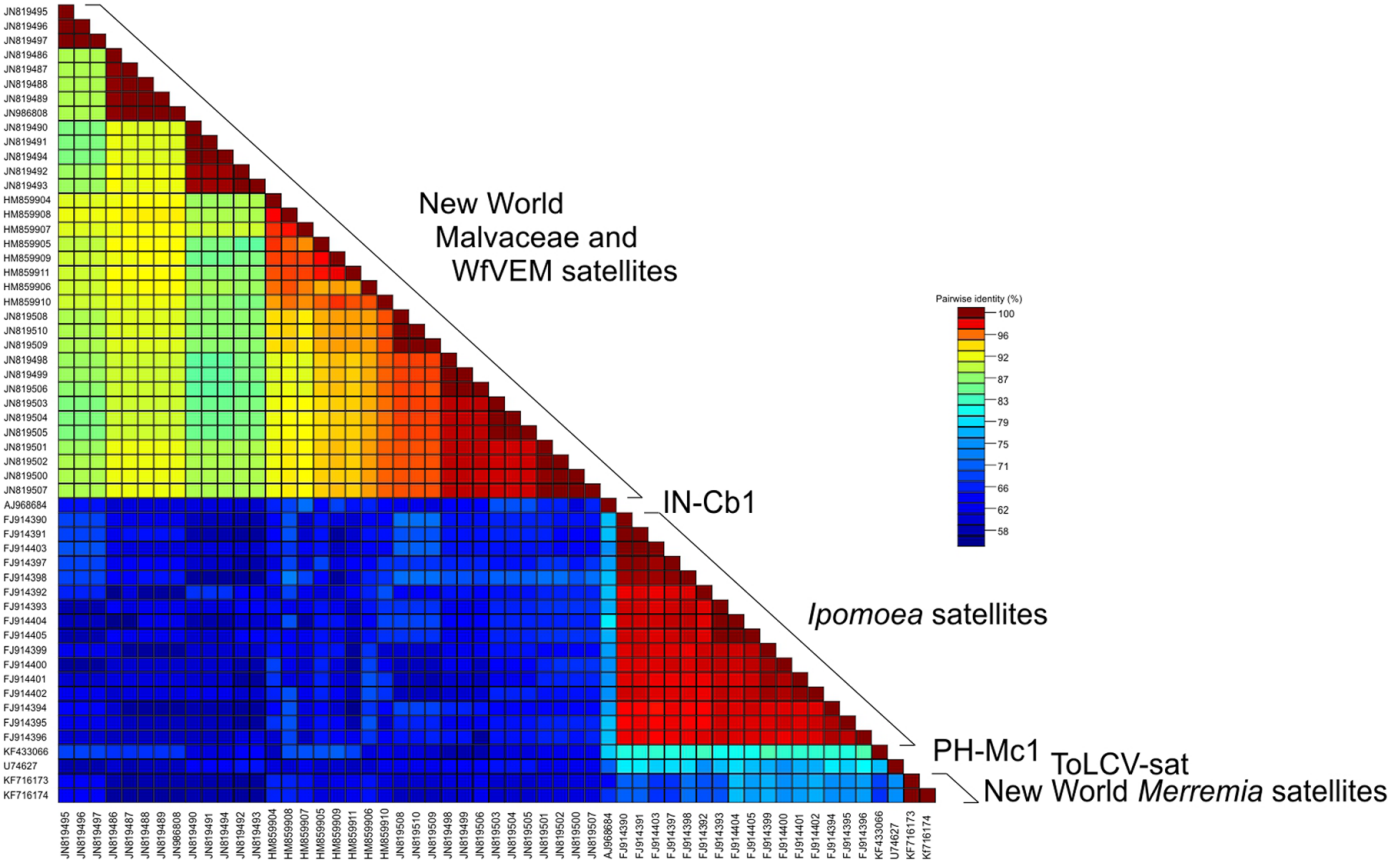
**Color pairwise nucleotide identity matrix of deltasatellites.***Ipomoea* and *Merremia* satellite sequences are described in this work and other sequences were retrieved from GenBank, including small satellites associated with New World begomoviruses infecting Malvaceae, ToLCV-sat, a satellite isolated from *C. bonplandianus* in India (IN-Cb1), and a satellite isolated from *M. coromandelianum* in Philippines (PH-Mc1). See **Table 2** and **Supplementary Table 1** for details on the compared sequences.

Grouping by geographic origin and plant species from which they were isolated is also evident for the satellites originating from Cuba, as noted previously (Fiallo-Olivé et al., 2012). This study also showed that the presumed begomovirus satellites isolated from *B. tabaci* insects collected in Florida (Ng et al., 2011) are closely related to the Cuban satellites and thus likely were associated with begomoviruses upon which the insects were feeding. The satellites isolated from whiteflies share relatively high levels of identity (76.2–94.8%) with the satellites isolated from *M. coromandelianum* and *S. micranthum* originating from Cuba (Fiallo-Olivé et al., 2012) but relatively low levels of identity (<66%) to all the other small satellites, including those from Venezuela characterized here (**Figure 6**; **Supplementary Table 2**). Nucleotide sequence comparisons between all small begomovirus-associated satellites showed them to form six distinct sub-groups: (i) Cuban and Florida satellites, (ii) IN-Cb1, (iii) *Merremia* satellites, (iv) ToLCV-sat, (v) PH-Mc1, and (vi) *Ipomoea* satellites. Nucleotide identity within the sub-groups range from 76 to 100% whereas identity between sub-groups range from 56 to 75% (**Figure 6**; **Supplementary Table 2**).

Although all the small satellites discussed here contain sequences homologous to the SCR of betasatellites, there are distinct differences between them. All the small satellites contain an insertion of ∼44 nt within the SCR-like sequences (relative to betasatellites; approximately coordinates 236–280 in the alignment shown in **Figure 2A**) between the nonanucleotide-containing hairpin structure and the first highly conserved (between all small satellites and betasatellites) sequence (approximately coordinates 210–235 in the alignment shown in **Figure 2A**). However, the sequences differ between the groups of satellites. The inserted sequence for WfVEM and the Cuban satellites has high levels of identity (>93%) but only low levels of identity (<46%) to the other small satellites, including those from Venezuela. For the satellites from Venezuela, isolated from *Merremia*, the insertion instead shows the highest levels of identity to ToLCV-sat (56.8%). For both IN-Cb1 and PH-Mc1 the insertion shows the highest levels of identity (59.1 and 63.6%, respectively) with the sweepovirus-associated satellites.

It is evident that, even amongst the betasatellites, there is considerable variation within the SCR. Nevertheless, for betasatellites, two blocks of conserved sequence can be distinguished (marked as blocks A and B in **Figure 2A**) with the sequence in block A (that contains the nonanucleotide-containing hairpin structure) more conserved than that in block B. The insertion in the small satellites is in block A, relative to betasatellites. However, for all small satellites originating from the NW (including those from *Merremia* characterized here), the insertion is smaller than for the small satellites from the OW (approximately coordinates 249–280 of the alignment in **Figure 2B**) and the inserted sequence differs between the *Merremia* (1764E13 and 1764E34) and other NW satellites (228H6, 177H1, VfVEM-Sat-g and VfVEM-Sat-a). For all satellites (including the betasatellites) block B is less well-conserved with the lowest levels of conservation for the satellites from the NW, particularly those isolated from the Malvaceae and whiteflies.

## Discussion

The study described here has identified small DNA satellites associated with sweepoviruses occurring in both the OW and NW (Lozano et al., 2009; Albuquerque et al., 2012). Although satellites from the two regions have related helper viruses, sweepoviruses, and infect related hosts (members of the genera *Ipomoea* and *Merremia*, both belonging to the family Convolvulaceae), they are genetically only distantly related. The structures of these DNA satellites share properties with other small non-coding DNA satellites identified in association with begomoviruses; ToLCV-sat, the first satellite identified in association with a DNA virus (Dry et al., 1997), satellites identified with bipartite NW begomoviruses infecting species of the Malvaceae in Cuba and satellites isolated from *B. tabaci* originating from Florida (Ng et al., 2011; Fiallo-Olivé et al., 2012).

Geminiviruses have a stringent size surveillance mechanism that operates during virus movement within the plant and there are also size constraints for encapsidation (Etessami et al., 1989; Elmer and Rogers, 1990; Frischmuth et al., 2001; Gilbertson et al., 2003). For defective interfering DNAs (diDNAs) associated with geminiviruses, which are typically half (∼1400 bp) the size of a begomovirus genome/DNA component, encapsidation has been shown to be in isometric particles (thus half a geminate particle; Frischmuth et al., 2001; Casado et al., 2004). Although betasatellites, which are typically 1350 bp in size, have been shown to be encapsidated in CP of the helper virus (Tabein et al., 2013) and insect transmissible (Saunders et al., 2000), the nature (multiplicity) of the particles has not been investigated. However, it would seem likely that, in common with diDNAs, they are encapsidated in isometric particles. The nature of virus particles encapsidating quarter unit-length DNAs, such as the ToLCV-sat-like satellites, remains unclear. However, encapsidation of ToLCV-sat DNA in the CP of ToLCV has been demonstrated by immunocapture PCR (Dry et al., 1997).

The sizes of the satellites characterized here range from 633 to 750. Thus, these molecules are about half the size of betasatellites and alphasatellites and about a quarter the size of begomovirus DNA components. The presence of an A-rich region is a common feature for all the DNA satellites associated with begomoviruses so far, including betasatellites, alphasatellites and the small non-coding satellites such as ToLCV-sat (Dry et al., 1997; Mansoor et al., 1999; Briddon et al., 2003; Fiallo-Olivé et al., 2012). For alphasatellites, it has been suggested that the A-rich region may be a “stuffer” required to increase the size of a nanovirus DNA component (∼1000 nt), from which alphasatellites are believed to have evolved, to that required for encapsidation by a begomovirus (∼1400 nt, about half the size of a begomovirus DNA component) (Mansoor et al., 2003). The betasatellites also have a size corresponding to half a begomovirus genome/DNA component with an A-rich region. By analogy to alphasatellites, this has been taken to indicate that betasatellites possible also have their origins as a component of another ssDNA virus, although no virus from which the component could have originated has yet been identified. Defective versions of betasatellites that are approximately half the size of full-length betasatellites are frequently identified in association with begomovirus-betasatellite infections (Briddon et al., 2003; Akhtar et al., 2014). Such defective molecules retain both the A-rich region and the SCR, but lack the βC1 coding sequence and resemble the satellite molecules identified in this study. Retention of the A-rich region and the SCR suggests that these are required for maintenance of these molecules by the helper begomovirus and thus is more than just a “stuffer,” since random mutation would otherwise rapidly reduce the A content. It has been suggested that the A-rich region may have a function in complementary-strand DNA replication, although evidence in support of this is lacking (Briddon et al., 2003).

For all the satellites discussed here, including the betasatellites, the presence of a second hairpin structure upstream of the SCR/SCR-like sequences appears to be a common feature; although for NW satellites the position differs from the remaining satellites (**Figures 1B** and **3C**). Only for some satellites (ToLCV-sat and some of the sweepovirus-associated satellites) is this structure also associated with sequences that match the predicted iteron sequence of the helper virus. Lin et al. (2003) showed that mutation of the “iteron” sequence in ToLCV-sat, which abolished interaction of the satellite with ToLCV Rep *in vitro*, did not abolish trans-replication of the satellite *in planta*. Additionally, ToLCV-sat could be trans-replicated and maintained in plants by other begomoviruses, and even a curtovirus, which do not have the same iterons as ToLCV (Dry et al., 1997). These findings suggest that, although the iteron sequence of ToLCV-sat may be important for interaction with ToLCV, other sequences, or possibly the hairpin structure, can act as a Rep binding site for both ToLCV and other viruses. Nawaz-ul-Rehman et al. (2009) put forward two hypotheses to explain the promiscuous interaction of betasatellites with begomoviruses. The “universal Rep” hypothesis proposed that begomoviruses that interact with betasatellites have Rep proteins with more relaxed origin recognition properties. The “universal iteron” hypothesis proposed that betasatellites contain sequences that allow them to be recognized by a greater range of Rep proteins (so-called “iteron-like” sequences). Although it remains unclear which hypothesis may be correct, the weight of evidence lies with the “universal iteron” hypothesis at this time. Saunders et al. (2008) showed that sequences important for trans-replication of betasatellites lie in the vicinity of the secondary hairpin and Nawaz-ul-Rehman et al. (2009) showed that, in adapting to a new helper begomovirus, these sequences may change to improve interaction with the virus. Also, in common with betasatellites, some of the small satellites have iteron-like sequences adjacent to the secondary hairpin structure (**Figures 3B,C**). This suggests that, due to long association with a single helper begomovirus, satellites such as ToLCV-sat and the sweepovirus-associated satellites have evolved a more close relationship with their helper viruses, by encoding helper virus iterons, but nevertheless maintain the iteron-like sequences of their betasatellites progenitors allowing them to interact with other begomoviruses (Saunders et al., 2002b; Lin et al., 2003).

It is evident based on the analyses presented here that the ToLCV-sat-like satellites are distinct from betasatellites. They lack the βC1 gene and have sequences that have diverged significantly from the SCR of betasatellites, including nonanucleotide sequence-containing hairpin loop structures that are distinct from those of betasatellites. Additionally they do not occur in the presence of betasatellites, unlike the defective betasatellite that have been identified in association with many betasatellites. On the basis of these findings it is proposed that the ToLCV-sat-like satellites be considered a class of satellites distinct from betasatellites, for which the name “deltasatellites” is proposed – the Greek letter delta often being used in molecular biology to indicate a deletion or mutation. The deltasatellites include the two groups of satellites identified here, the satellites associated with sweepoviruses infecting *Ipomoea* sp. in Spain and *Merremia* in Venezuela, ToLCV-sat, the small molecules identified in *Croton* from India and *Malvastrum* from the Philippines, the satellites identified by Fiallo-Olivé et al. (2012) isolated from *Malvastrum* and *Sidastrum*, as well as a group of small molecules identified in *B. tabaci* whiteflies originating from the United States of America (Ng et al., 2011).

Although the deltasatellites and betasatellites are clearly related, it is unclear whether one group evolved from the other or they have distinct origins. Based on the premise that the simplest explanation is frequently the correct one, it would seem most likely that the delatasatellites evolved from the betasatellites. This possibility is supported by the apparent ease with which betasatellites lose their βC1 coding sequence (Briddon et al., 2003; Akhtar et al., 2014). Thus a betasatellite would have lost its βC1 gene, becoming approximately one quarter the size of a begomovirus genome/genomic component, and the SCR-derived sequences diverged from the parent betasatellite once the betasatellite was lost by the virus. What forces might have driven the divergence of the SCR sequences is unclear since the precise function of these sequences remains unknown. Since, the SCR occupies a position analogous to the common region shared by components of bipartite begomoviruses, which ensure the integrity of the split genome by virtue of encompassing the *ori*, it has been suggested that the SCR sequences may be involved in transreplication by the helper begomovirus (Briddon et al., 2003). However, for transreplication of betasatellites, specificity has been shown to be mediated by sequences between the A-rich region and the SCR (Saunders et al., 2008). If the SCR is involved in interactions with the helper begomoviruses, the divergence of the SCR sequences of deltasatellites might have been driven by the need to interact with the helper begomovirus.

The fact that the nonanucleotide-containing hairpin structures of delatasatellites differ from those of betasatellites suggests that the betasatellite(s) from which deltasatellites evolved has yet to be identified or is extinct. Additionally, the difference in the nonanucleotide-containing hairpin structures between distinct deltasatellites that each lineage has a distinct origin (convergent evolution). The identification of deltasatellites in a vegetatively propagated species provides a possible explanation of the present wide geographic distribution of these satellites. Sweet potato has its evolutionary origins in tropical South and Central America and has been transported to all the warmer parts of the World for cultivation (Roullier et al., 2013).This is not, however, evidence for a NW origin of deltasatellites since sweet potato germplasm has been moved both to and from its geographic origin (Paprotka et al., 2010a) and the sweepoviruses are typical OW begomoviruses (Brown et al., 2012). Further deltasatellites will need to be identified and characterized to more precisely determine the origins and evolution of these satellites.

Despite the fact that ToLCV-sat was identified in the 1990s (Dry et al., 1997), little is known concerning the maintenance of deltasatellites by begomoviruses and the effects they have on virus infections. So far only ToLCV-sat has been experimentally introduced into plants and these studies did not look at the effects of the satellite on symptoms and virus replication (Dry et al., 1997; Lin et al., 2003). There is thus a need to investigate the effects of deltasatellites on begomovirus infections and their interactions with helper begomoviruses, for comparison to the better characterized, and more numerous, betasatellites and alphasatellites. These aspects will be the focus of future studies.

## Author Contributions

JN-C designed and supervised the study. DC and FG-P provided and identified plant samples. GL, HT, and EF-O performed the experiments and bioinformatic analyses. JN-C and RWB performed bioinformatic analyses and wrote the main manuscript text. All authors reviewed the manuscript.

## Conflict of Interest Statement

The authors declare that the research was conducted in the absence of any commercial or financial relationships that could be construed as a potential conflict of interest.

## Footnotes

1 http://www.ncbi.nlm.nih.gov/

2 http://molbiol-tools.ca/Jie_Zheng/dna.html

3 http://www.bioinformatics.org/sms2/shuffle_dna.html
