# Supplementary material for: Characterization of Non-coding DNA Satellites Associated with Sweepoviruses (Genus Begomovirus, Geminiviridae) – Definition of a Distinct Class of Begomovirus-Associated Satellites
Source: Front Microbiol. 2016 Feb 17;7:162. doi: 10.3389/fmicb.2016.00162 (PMC4756297; doi:10.3389/fmicb.2016.00162)
Supplement: Supplementary file 1 [file Table_1.DOCX]

**SUPPLEMENTARY TABLE 1 |** Sequences of DNA satellites available in GenBank used in the analyses carried out in this work.

| DNA satellite | GenBank acc. no. | Reference |
| --- | --- | --- |
| ToLCV-sat | U74627 | 1 |
| *M. coromandelianum* 177H1 | JN986808 | 2 |
| *M. coromandelianum* 177H3 | JN819486 | 2 |
| *M. coromandelianum 177N2* | JN819487 | 2 |
| *M. coromandelianum* 177N4 | JN819488 | 2 |
| *M. coromandelianum* 177N7 | JN819489 | 2 |
| *M. coromandelianum* 228H1 | JN819490 | 2 |
| *M. coromandelianum* 228H3 | JN819491 | 2 |
| *M. coromandelianum* 228H5 | JN819492 | 2 |
| *M. coromandelianum* 228H6 | JN819493 | 2 |
| *M. coromandelianum* 228H6B | JN819494 | 2 |
| *M. coromandelianum* 412N1 | JN819498 | 2 |
| *M. coromandelianum* 412N2 | JN819506 | 2 |
| *M. coromandelianum* 412N3 | JN819499 | 2 |
| *M. coromandelianum* 413N1 | JN819507 | 2 |
| *M. coromandelianum* 413N2 | JN819500 | 2 |
| *M. coromandelianum* 413N3 | JN819501 | 2 |
| *M. coromandelianum* 413N5 | JN819502 | 2 |
| *M. coromandelianum* 414N1 | JN819503 | 2 |
| *M. coromandelianum* 414N2 | JN819504 | 2 |
| *M. coromandelianum* 414N3 | JN819505 | 2 |
| *M. coromandelianum* 424N1 | JN819508 | 2 |
| *M. coromandelianum* 424N2 | JN819509 | 2 |
| *M. coromandelianum* 424N3 | JN819510 | 2 |
| *S. micranthum* 404N1 | JN819495 | 2 |
| *S. micranthum* 404N2 | JN819496 | 2 |
| *S. micranthum* 404N3 | JN819497 | 2 |
| WfVEM-Sat-a | HM859908 | 3 |
| WfVEM-Sat-b | HM859906 | 3 |
| WfVEM-Sat-c | HM859907 | 3 |
| WfVEM-Sat-d | HM859905 | 3 |
| WfVEM-Sat-e | HM859904 | 3 |
| WfVEM-Sat-f | HM859911 | 3 |
| WfVEM-Sat-g | HM859910 | 3 |
| WfVEM-Sat-h | HM859909 | 3 |
| *M. coromandelianum* (PH-Mc1)  *C. bonplandianus*  (IN-Cb1) | KF433066  AJ968684 | unpublished  unpublished |
| 1, Dry et al. (1997) | | |
| 2, Fiallo-Olivé et al. (2012) | | |
| 3, Ng et al. (2011) | | |
