# Supplementary material for: Characterization of Non-coding DNA Satellites Associated with Sweepoviruses (Genus Begomovirus, Geminiviridae) – Definition of a Distinct Class of Begomovirus-Associated Satellites
Source: Front Microbiol. 2016 Feb 17;7:162. doi: 10.3389/fmicb.2016.00162 (PMC4756297; doi:10.3389/fmicb.2016.00162)
Supplement: Supplementary file 2 [file Table_2.DOCX]

**SUPPLEMENTARY TABLE 2 |** Percentage nucleotide sequence identities between small begomovirus-associated satellites.

|  | *Ipomoea* | *Merremia* | ToLCV-sat | *Malvastrum* | *Sidastrum* | WfVEM | IN-Cb1 | PH-Mc1 |
| --- | --- | --- | --- | --- | --- | --- | --- | --- |
| *Ipomoea*  (16) | 95-100 |  |  |  |  |  |  |  |
| *Merremia*  (2) | 62-68 | 100 |  |  |  |  |  |  |
| ToLCV-sat  (1) | 67-71 | 67 | 100 |  |  |  |  |  |
| *Malvastrum*  (23) | 56-66 | 56-60 | 57-61 | 76-100 |  |  |  |  |
| *Sidastrum*  (3) | 56-64 | 61 | 61 | 77-80 | 100 |  |  |  |
| WfVEM  (8) | 58-66 | 59-62 | 58-62 | 76-95 | 80-83 | 88-100 |  |  |
| IN-Cb1  (1) | 67-71 | 61-63 | 65 | 59-64 | 61 | 60-65 | 100 |  |
| PH-Mc1  (1) | 73-75 | 63 | 69 | 57-63 | 63 | 57-65 | 69 | 100 |

The numbers in brackets indicate the numbers of sequences compared. Percentage nucleotide sequence identities were calculated using the Sequence Demarcation Tool (SDT) that ignores gaps (Muhire et al., 2014). Values higher than 75% are highlighted in grey.
